# Systemic inflammatory regulators and risk of acute-on-chronic liver failure: A bidirectional mendelian-randomization study

**DOI:** 10.3389/fcell.2023.1125233

**Published:** 2023-01-19

**Authors:** Shengnan Wang, Hao Zhu, Lin Pan, Mengyuan Zhang, Xiaoqiang Wan, Hongqin Xu, Rui Hua, Mingqin Zhu, Pujun Gao

**Affiliations:** ^1^ Department of Neurology, The First Hospital of Jilin University, Changchun, China; ^2^ Department of Hepatology, The First Hospital of Jilin University, Changchun, China; ^3^ Clinical College, Jilin University, Changchun, China; ^4^ Department of Respiratory, The First Hospital of Jilin University, Changchun, China; ^5^ Department of Interventional Radiology, The First Hospital of Jilin University, Changchun, China

**Keywords:** acute-on-chronic liver failure, inflammation, mendelian randomization, cytokines, IL-13

## Abstract

Inflammation plays a role in the pathogenesis of acute-on-chronic liver failure (ACLF), however, whether there is a causal relationship between inflammation and ACLF remains unclear. A two-sample Mendelian randomization (MR) approach was used to investigate the causal relationship between systemic inflammatory regulators and ACLF. The study analyzed 41 cytokines and growth factors from 8,293 individuals extracted from a genome-wide association study (GWAS) meta-analysis database involving 253 ACLF cases and 456,095 controls. Our results showed that lower stem cell factor (SCF) levels, lower basic fibroblast growth factor (bFGF) levels and higher Interleukin-13 (IL-13) levels were associated with an increased risk of ACLF (OR = 0.486, 95% CI = 0.264–0.892, *p* = 0.020; OR = 0.323, 95% CI = 0.107–0.972, *p* = 0.044; OR = 1.492, 95% CI = 1.111–2.004, *p* = 0.008, respectively). In addition, genetically predicted ACLF did not affect the expression of systemic inflammatory regulators. Our results indicate that cytokines play a crucial role in the pathogenesis of ACLF. Further studies are needed to determine whether these biomarkers can be used to prevent and treat ACLF.

## 1 Introduction

Acute-on-chronic liver failure (ACLF) is a distinct clinical syndrome characterized by chronic liver disease and acute hepatic injury (Sarin and Choudhury, 2016). The incidence of ACLF is increasing due to more alcohol consumption, drug use, and the growing number of obese and diabetic patients (Byass, 2014). ACLF is characterized by persistent inflammation, immune dysregulation, and widespread immune activation (Tao et al., 2022). The systemic inflammatory response syndrome is characterized by a suppressed immune system and a subsequent sepsis (Sarin and Choudhury, 2016). It has been suggested that pathogens are not adequately responded to advanced cirrhosis and ACLF, because of an acquired alteration of the innate immune system, which ultimately results in multiple organ failure and a higher death rate (Wasmuth et al., 2005).

ACLF could benefit from anti-inflammatory intervention targeting cytokines which have crucial roles in pathogenesis of inflammation (Ouyang et al., 2011; Mantovani et al., 2019). The association between circulating levels of inflammatory cytokines and risk of ACLF has only been investigated in few studies. It was reported that patients with ACLF had higher white blood cell (WBC) and C-reactive protein (CRP) levels than those with decompensated cirrhosis without ACLF (Dirchwolf et al., 2016). In a study by Mehta et al., TNF-a, IL-8, and IL-6 levels were found significantly higher among ACLF patients (Mehta et al., 2015). Hepatitis B-related ACLF patients had high levels of serum IL-12 and IL-21, according to a recent study (Du et al., 2021). Reverse causation and residual confounding are common biases in conventional observational studies (Smith and Ebrahim, 2003), therefore, we need to determine whether these changes in inflammatory regulators cause inflammation or they are a response to inflammation (Tamburini et al., 2019). There is still a great deal of uncertainty regarding the potential causal role of individual cytokines in determining ACLF. It would be useful to discover biomarkers for early diagnosis and develop prevention strategies by understanding the pathophysiological mechanisms.

By using genetic variants as instrument variables (IVs) of modifiable exposures, mendelian randomization (MR) studies can overcome the limitations of observational studies (Smith and Ebrahim, 2003). It allows investigation of associations independent of conventional biases associated with observational studies by using genetic variants as IVs (Holmes et al., 2017). To assess the causal relationship between inflammatory cytokines and ACLF risk, we used the MR method in this study. Furthermore, systemic inflammatory regulators were evaluated in association with genetically predicted ACLF.

## 2 Methods

### 2.1 Study design

**FIGURE 1:**
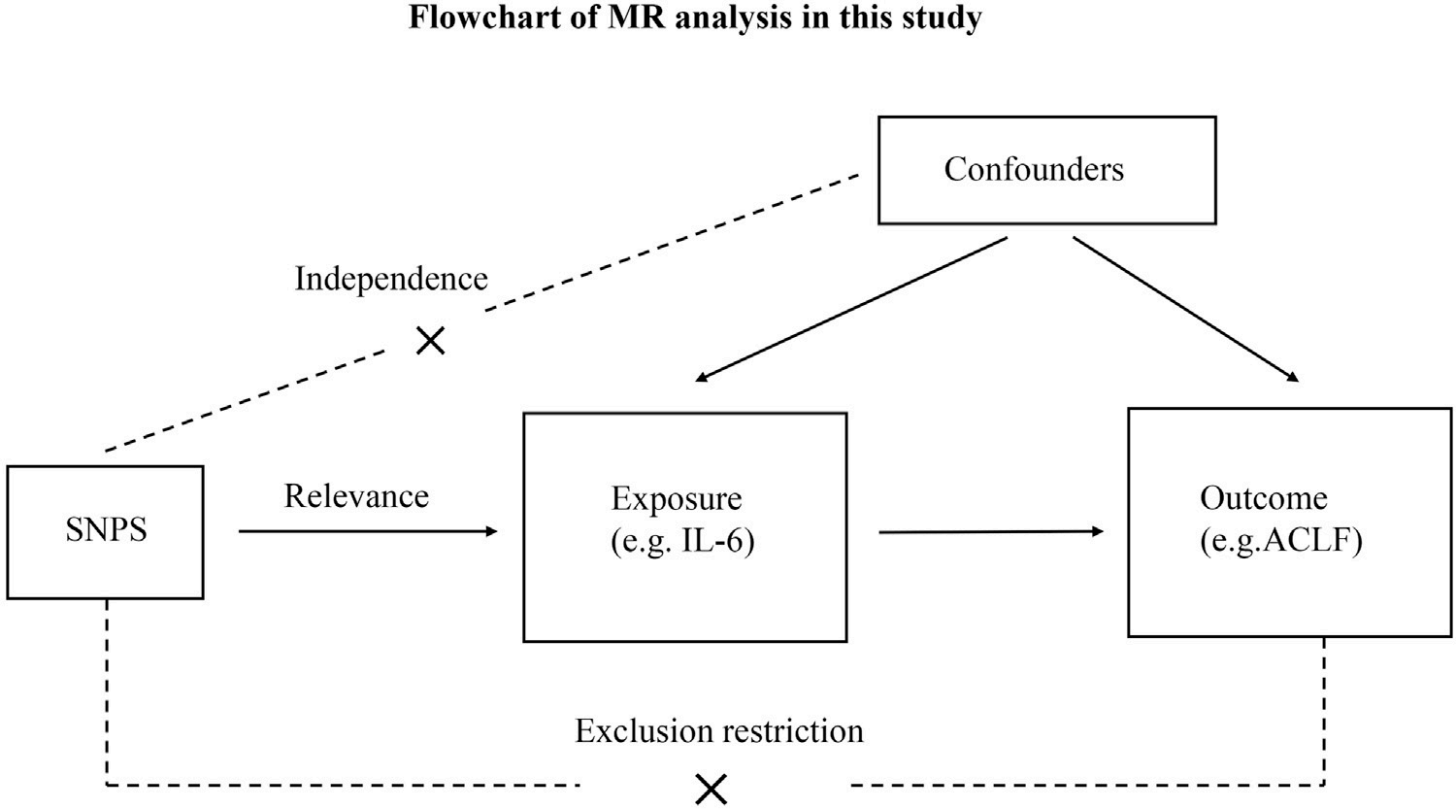
Flowchart of MR analysis in this study. ACLF, Acute-on-chronic liver failure; SNPs, single-nucleotide polymorphisms.

Figure 1 shows the overall design of the present study. Because we used summary statistics from published studies, we did not need to obtain any additional ethical approvals. There are three critical assumptions in MR analysis: (i) Exposure is strongly associated with IVs; (ii) Exposure and outcome confounders should not affect IVs; and (iii) Exposure is the only factor that mediates IV-outcome associations (Smith and Ebrahim, 2003).

### 2.2 The selection of data sources and instruments

Table 1 summarizes details about the MR analyses based on summary-level data from genome-wide association studies (GWAS) about cytokines. The full GWAS summary statistics are available on the GWAS Catalog server at https://www.ebi.ac.uk/gwas/home. Data about circulating cytokines were gathered from a study of 8,293 individuals that included 41 cytokines and growth factors (Ahola-Olli et al., 2017). We extracted the summary GWAS statistics of ACLF from the United Kingdom Biobank, which included 253 ACLF cases and 456,095 controls of European ancestry, and a generalized linear mixed model (GLMM)-based method named (fastGWA-GLMM) was utilized with adjustments for covariates. ACLF was defined according to Asia–Pacific Association for the Study of the Liver (APASL) criteria: patients with previously diagnosed or undiagnosed chronic liver disease associated with high mortality had acute liver injury, characterized by jaundice and coagulation dysfunction, and ascites and/or encephalopathy within 4 weeks (Sarin et al., 2019). Acute insults include alcohol, hepatotropic viruses and drugs whereas the underlying chronic liver disease is generally cirrhosis due to alcohol, hepatitis B or C, or non-alcoholic steatohepatitis (Jiang et al., 2021). Additionally, we used a genome-wide threshold of significance (*p* < 5 × 10^–8^) to avoid selecting false positive instruments. In linkage disequilibrium, single nucleotide polymorphisms (SNP) with the lowest *p*-value were retained as independent, after pruning all SNPs in linkage disequilibrium (LD; *r* ^2^ < 0.01 in the European 1000G reference panel) (Georgakis et al., 2021). Following harmonization of the selected SNPs with outcome data, only 10 cytokines had more than two independent SNPs at *p* < 5 × 10^–8^ level (Supplementary Table S1). We therefore adopted an alternative threshold of *p* < 5 × 10^−6^. Under these conditions, we chose all 41 systemic inflammation regulators. (Supplementary Table S2). The F-statistics of SNPs were averaged to avoid weak IVs, with IVs with F-statistics over 10 considered strong (Bowden et al., 2016).

**TABLE 1:** The sample size for each cytokine analyzed in this study acquired from the GWAS.

Cytokines	Abbreviation	Sample size	Number
Cutaneous T cell attracting (CCL27)	CTACK	3,631	GCST004420
Beta nerve growth factor	βNGF	3,531	GCST004421
Vascular endothelial growth factor	VEGF	7,118	GCST004422
Macrophage migration inhibitory factor (glycosylation-inhibiting factor)	MIF	3,494	GCST004423
TNF-related apoptosis inducing ligand	TRAIL	8,186	GCST004424
Tumor necrosis factor-beta	TNFβ	1,559	GCST004425
Tumor necrosis factor-alpha	TNFα	3,454	GCST004426
Stromal cell-derived factor-1 alpha (CXCL12)	SDF1α	5,998	GCST004427
Stem cell growth factor beta	SCGFβ	3,682	GCST004428
Stem cell factor	SCF	8,290	GCST004429
Interleukin-16	IL-16	3,483	GCST004430
Regulated on Activation, Normal T cell Expressed and Secreted (CCL5)	RANTES	3,421	GCST004431
Platelet derived growth factor BB	PDGFbb	8,293	GCST004432
Macrophage inflammatory protein-1β (CCL4)	MIP1β	8,243	GCST004433
Macrophage inflammatory protein-1α (CCL3)	MIP1α	3,522	GCST004434
Monokine induced by interferon-gamma (CXCL9)	MIG	3,685	GCST004435
Macrophage colony-stimulating factor	MCSF	840	GCST004436
Monocyte specific chemokine 3 (CCL7)	MCP3	843	GCST004437
Monocyte chemotactic protein-1 (CCL2)	MCP1	8,293	GCST004438
Interleukin-12p70	IL-12p70	8,270	GCST004439
Interferon gamma-induced protein 10 (CXCL10)	IP10	3,685	GCST004440
Interleukin-18	IL-18	3,636	GCST004441
Interleukin-17	IL-17	7,760	GCST004442
Interleukin-13	IL-13	3,557	GCST004443
Interleukin-10	IL-10	7,681	GCST004444
Interleukin-8 (CXCL8)	IL-8	3,526	GCST004445
Interleukin-6	IL-6	8,189	GCST004446
Interleukin-1 receptor antagonist	IL1ra	3,638	GCST004447
Interleukin-1-beta	IL-1β	3,309	GCST004448
Hepatocyte growth factor	HGF	8,292	GCST004449
Interleukin-9	IL-9	3,634	GCST004450
Interleukin-7	IL-7	3,409	GCST004451
Interleukin-5	IL-5	3,364	GCST004452
Interleukin-4	IL-4	8,124	GCST004453
Interleukin-2 receptor, alpha subunit	IL2rα	3,677	GCST004454
Interleukin-2	IL-2	3,475	GCST004455
Interferon-gamma	IFN-γ	7,701	GCST004456
Growth regulated oncogene-α (CXCL1)	GROα	3,505	GCST004457
Granulocyte colony-stimulating factor	GCSF	7,904	GCST004458
Basic fibroblast growth factor	bFGF	7,565	GCST004459
Eotaxin (CCL11)	Eotaxin	8,153	GCST004460

Considering the smaller sample size of the ACLF trait, the *p*-value of exposure IVs was set at 5 × 10^–6^ to investigate the causal effect of ACLF on systemic inflammatory regulators. In Supplementary Table S3, selected SNPs are listed along with selection procedures for systemic inflammatory regulators.

### 2.3 Statistical analysis

Inverse variance weighted (IVW) analysis was used as the main MR analysis. The Cocrane’s Q test was used to determine whether SNPs were heterogeneous (Song et al., 2022). In complementary analyses, weighted median and MR-Egger regression methods were used (Larsson et al., 2017). A measure of directional pleiotropy was derived from the intercept obtained from MR-Egger regression (*p* < 0.05 was considered significant) (Bowden et al., 2015), and MR-PRESSO was used to test for outlier SNPs (Verbanck et al., 2018). We performed “leave-one-out” analyses which excluded one SNP at a time to test the stability of our results. If the IVW method result is significant (*p* < 0.05), even if the results of other methods are not significant, and no pleiotropy and heterogeneity was identified, it can be regarded as a positive result, provided that the beta values of the other methods are in the same direction (Chen et al., 2020). If horizontal pleiotropy was identified but no heterogeneity, the MR-Egger method will be selected. If heterogeneity was identified but no pleiotropy, the weight median method was selected, or the multiplicative random-effects inverse variance weighting (mre-IVW) method was used for analysis. The analyses were conducted using the packages TwoSampleMR (version 0.5.6) and MRPRESSO (version 1.0) in R (version 4.2.1). The results of pleiotropy, heterogeneity, and sensitivity analysis can be found in Supplementary Tables S2, S3.

## 3 Results

As demonstrated by the following results, genetic predicted systemic inflammatory regulators are associated with ACLF. The higher stem cell factor (SCF) levels and basic fibroblast growth factor (bFGF) levels are inversely associated with decreased risks of ACLF (OR = 0.486, 95% CI = 0.264–0.892, *p* = 0.020; OR = 0.323, 95% CI = 0.107–0.972, *p* = 0.044, respectively) using IVW methods. MR-Egger Intercept did not detect potential horizontal pleiotropy for SCF and bFGF (*p* = 0.790; *p* = 0.898, respectively). Furthermore, Q values based on MR-Egger and IVW tests showed that there was no obvious heterogeneity for SCF and bFGF (all *p* values > 0.05).

The higher circulating level of Interleukin-13 (IL-13) was found to be related to an increased risk of ACLF (OR = 1.492, 95% CI = 1.111–2.004, *p* = 0.008, respectively) using IVW methods. MR-Egger Intercept did not detect potential horizontal pleiotropy for IL-13 (*p* = 0.287, respectively). Furthermore, there was also no obvious heterogeneity for IL-13 (all *p* values > 0.05). Leave-one-out studies were used for sensitivity analysis and demonstrated no influence of individual studies.

**FIGURE 2:**
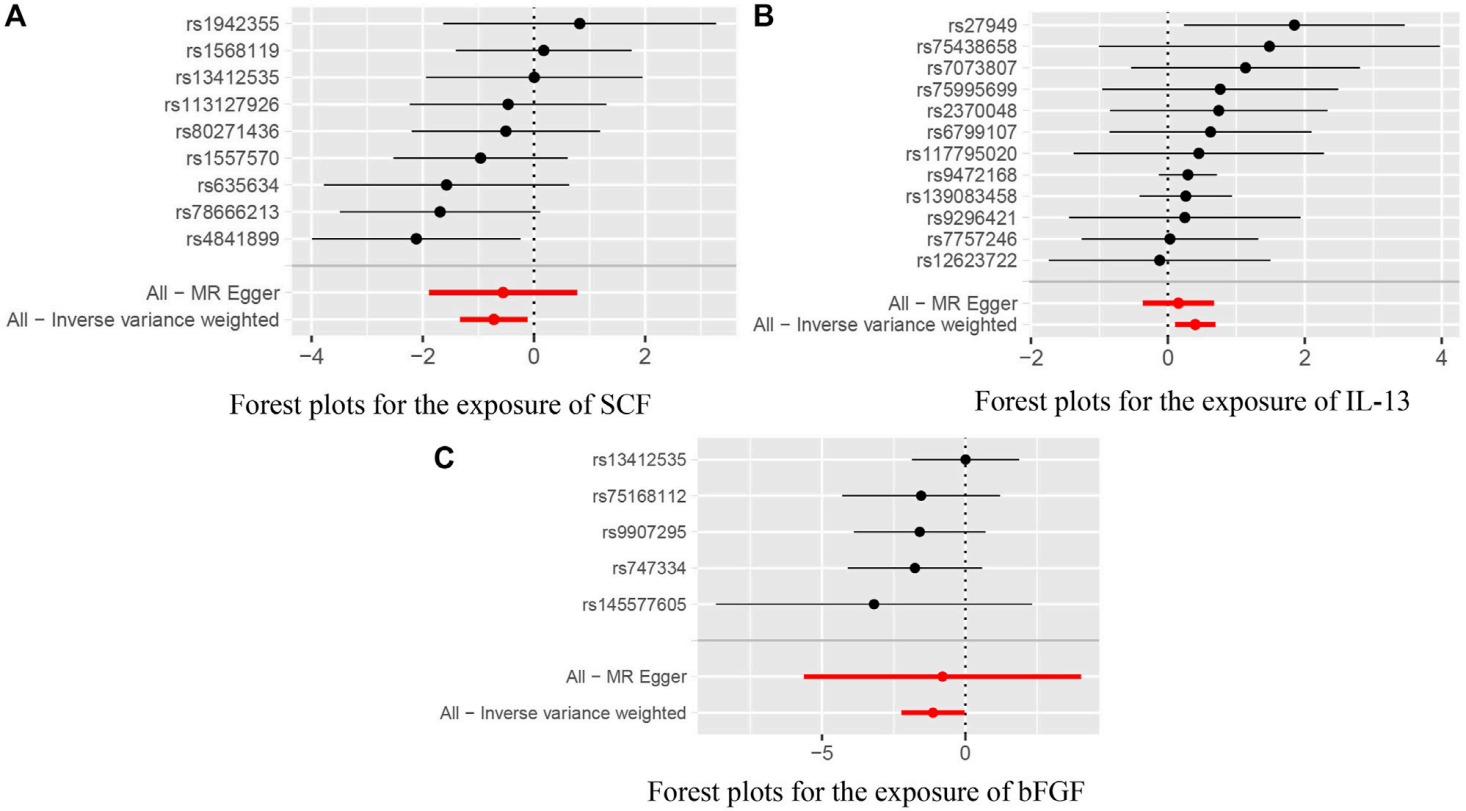
**(A)** Forest plots for the exposure of SCF. **(B)** Forest plots for the exposure of IL-13. **(C)** Forest plots for the exposure of bFGF.

In addition, we identified suggestive associations between genetically predicted macrophage inflammatory protein 1a levels and ACLF risk (OR = 1.870, 95% CI = 1.077–3.247, *p* = 0.026, respectively) using IVW methods. The evidence of pleiotropy was still observed using the MR-PRESSO global test (*p* < 0.01). Horizontal pleiotropy could violate MR assumptions, so this analysis might not be reliable. The above results are presented in Table 2. Genetically predicted systemic inflammatory regulators and ACLF risk analysis methods MR Results, heterogeneity analysis, pleiotropy analysis and sensitivity analysis results are summarized in Supplementary Table S2. Figure 2 shows forest plots of the above results. The scatter plots, funnel plots, and leave-one-out plots are summarized in Supplementary Figures S1–S3.

**TABLE 2:** Results of the MR study testing causal association between systemic inflammatory regulators and risk of ACLF.

Cytokines	Number of SNPs	Beta	Or (95% CI)	P	P For heterogeneity test	P For MR-Egger intercept	P For MR-PRESSO (0 outliers)
**Stem cell factor**							
Inverse variance weighted	9	−0.722	0.486 (0.264–0.892)	0.020	0.501	0.790	0.179
MR Egger	9	−0.555	0.574 (0.151–2.178)	0.441	0.403		
Weighted median	9	−0.503	0.605 (0.263–1.392)	0.237			
**IL-13**							
Inverse variance weighted	12	0.400	1.492 (1.111–1.204)	0.008	0.862	0.287	0.081
MR Egger	12	0.154	1.167 (0.693–1.694)	0.574	0.897		
Weighted median	12	0.288	1.333 (0.923–1.926)	0.125			

Cytokines	Number of SNPs	Beta	Or (95% CI)	P	P For heterogeneity test	P For MR-Egger intercept	P For MR-PRESSO
**Basic fibroblast growth factor**							
Inverse variance weighted	5	−1.130	0.323 (0.107–0.972)	0.044	0.649	0.900	0.105
MR Egger	5	−0.799	0.450 (0.004–56.475)	0.767	0.483		
Weighted median	5	−1.558	0.211 (0.051–0.871)	0.031			
**Macrophage inflammatory protein-1α (CCL3)**							
Inverse variance weighted	8	0.626	1.870 (1.077–3.247)	0.026	0.848	0.964	0.010
MR Egger	8	0.592	1.807 (0.398–8.203)	0.473	0.760		
Weighted median	8	0.443	1.558 (0.759–3.198)	0.227			

We predicted the association between genetically predicted ACLF and cytokine levels with the same approach. However, genetically predicted ACLF was not associated with any cytokine levels in any MR methods. The results of MR, heterogeneity, pleiotropy and sensitivity analysis of all methods related to the analysis of genetically predicted ACLF and cytokine levels are summarized in Supplementary Table S3.

## 4 Discussion

Clinicians and researchers are becoming increasingly interested in ACLF, a syndrome occurring in patients with chronic liver disease, particularly cirrhosis. ACLF is a high mortality disease characterized by failure of different organs and systems (Jalan et al., 2014; Arroyo et al., 2015). Inflammation plays a significant role in the pathogenesis and progression of ACLF (Bernal et al., 2015). The immunological defects in ACLF patients are similar to those in sepsis patients (Wan et al., 2013). Numerous studies have shown that immune cell fractions vary significantly during ACLF development (Hassan et al., 2022). The inflammatory cytokine pathways (mediators) will be activated and lead to tissue injury. Dying parenchymal and non-parenchymal cells release damage associated molecular patterns (DAMPs) and other cytokines (Medzhitov, 2010). There is still a failure in preventative and therapeutic interventions for ACLF, and we do not yet fully understand ACLF etiology and pathogenesis. It is crucial to unravel the pathophysiological pathway in advanced cirrhosis and ACLF.

By examining 41 cytokines in the largest GWAS datasets available, we found that lifelong elevated levels of IL-13 may increase ACLF risk, and elevated circulating SCF and bFGF levels could decrease risk of ACLF. To our knowledge, this is the largest and most comprehensive MR study to date to explore genetically inflammatory cytokines’ association with ACLF.

IL-13 is a Th2 cytokine produced by Th2-polarized cells (Nam et al., 2008) that is associated with allergic diseases, asthma, and tissue fibrosis (Passalacqua et al., 2017). Only a few observational studies have shown an association between IL-13 and ACLF risk over the past few decades. There is a strong IL-13 response in animal models during the acute liver injury progression (Li et al., 2019), suggesting that IL-13 might be involved in the development of liver injury. In hepatic encephalopathy (HE) patients, inflammation and cognition may be synergistic due to IL-13’s high correlation with gut microbiome components (Keshavarzian et al., 1999). In cirrhotic patients with HE, IL-13 concentrations in the patients were higher, even though no allergic disorders were present (Marra and Annunziato, 2010). Therefore, IL-13 may contribute to ACLF initiation and progression.

Observational studies may be biased due to their small sample size and potential confounding factors. Based on the results of this MR analysis, we were able to reach a reliable conclusion that increased circulating IL-13 levels was associated with a higher risk of ACLF (OR = 1.492, 95% CI = 1.111–2.004, *p* = 0.008). It appears that lowering IL-13 levels may be an effective therapeutic approach for ACLF.

The following pathways may explain how IL-13 contributes to ALCF pathophysiology. Hepatic fibrosis is mainly caused by IL-13, which has been shown to play a major role in its development (Caldas et al., 2008). Inhibiting IL-13 activity with IL-13Rα2 can reduce liver fibrosis (Lee et al., 2010). As a result of liver injury, hepatocellular IL-33 is secreted, which stimulates ILC2 to produce IL-13 (Seki and Schwabe, 2015). IL-13 is also involved in mediating allergic reactions, suggesting the profibrotic potential of IL-13 and widespread immunomodulatory disturbances that are prevalent in cirrhosis.

bFGF plays an important role in various diseases, including angiogenesis, myocardial ischemia, and spinal cord injury (Wang et al., 2015; Elbialy et al., 2021; Zhu et al., 2021). A study has suggested that bFGF-knockout mice heal full-thickness wounds more slowly than wild-type mice (Abdelhakim et al., 2020). Experimental studies have demonstrated that liver failure and related liver diseases can be treated with encapsulated hepatocytes and hFLSCs/bFGF transplantation (Wilson et al., 2011). The statistical analyses showed an inverse association between increased circulating bFGF levels and lower risks of ACLF (OR = 0.323, 95% CI = 0.107–0.972, *p* = 0.044). According to the Nrf2/Hippo signaling pathway, bFGF could reduce liver ischemia-reperfusion injury and hepatocyte injury (Chen et al., 2022a). As well as initiating liver development (Jung et al., 1999), bFGF can also promote the trans differentiation of bone marrow cells into hepatic lineage cells *in vitro* (Saji et al., 2004; Kamada et al., 2009). As a result of these findings, bFGF appears to be a potential therapeutic approach for ACLF.

SCF is a cytokine found in hematopoietic stem cells (HSCs) that promotes proliferation and differentiation of cells in response to its receptor (c-kit) (Khodadi et al., 2016). The use of stem cells as a potential alternative treatment for liver disease is on the rise. Several studies have suggested SCF plays an important role in the remarkable phenomenon of cell proliferation and compensatory growth after liver injury (Swenson et al., 2008; Liau et al., 2018). A recent study demonstrated a significant reduction in serum SCF levels in patients with fulminant hepatitis with a poor prognosis (Okumoto et al., 2007). The statistical analyses showed an inverse association between increased circulating SCF levels and lower risks of ACLF (OR = 0.486, 95% CI = 0.264–0.892, *p* = 0.020). Our findings are in line with results from studies in recent years (ref). SCF can protect the liver cells by recruiting inflammatory cells, stimulating hepatobiliary cell proliferation, guiding cell migration, and inducing vascularization to restore tissue integrity (Meng et al., 2012). SCF is a natural ligand of the c-kit receptor. The expression of c-kit, the SCF receptor, increases in patients with fulminant hepatic failure, suggesting a role for both SCF and c-kit. It is important to investigate further the mechanisms that inhibits ACLF in the future.

Furthermore, bidirectional MR analysis revealed that ACLF may not be associated with changes in cytokine levels in the blood. Several observational studies have explored the role of cytokines in liver failure. The serum levels of CRP, IL-1β and IL-6 are higher in patients with ACLF (Liu et al., 2014; Xiao et al., 2020). An additional study found that IL-10, IL-7, MCP-1, IL-12, TNF-α, IFN-γ, and G-CSF levels were significantly decreased in ACLF patients, which is consistent with an inappropriate immune response (Dirchwolf et al., 2016). Some studies believe that there is an abnormal plasma cytokine profile associated with ACLF syndrome, mainly related to chemotaxis and migration of leukocytes (Solé et al., 2016). And others think that in patients with decompensated cirrhosis and ACLF, the immune dysfunction associated with cirrhosis has been proposed to switch from a pro-inflammatory phenotype to an immunodeficient phenotype (Dirchwolf et al., 2016). The findings may be influenced by residual measured and unmeasured confounding. There is a possibility that many other factors influence cytokine production, including the cytokine network system rather than the disease itself.

However, our study has several limitations. First, to extract SNPs from GWAS data on cytokines, we used a significance cut-off of *p* < 5 × 10^−6^ as with *p* < 5 × 10^−8^ as the cut-off value, only 10 had at least one genome-wide significant SNP. Second, our MR-Egger and Weight Median estimates were not significant. Of course, it is best if all three methods have significant results. The IVW approach, however, has a significantly higher statistical power than the other MR approaches, especially MR-Egger (Lin et al., 2021). Researchers have also strengthened the requirement that MR approaches follow a consistent beta direction, which we used in our study as well (Bowden et al., 2016; Venkatesh et al., 2022). Therefore, as long as the results of IVW method are significant and those of other methods are not, but the beta direction is the same, they can also be considered as significant results (Chen et al., 2022b). The third issue is that all GWAS data came from European populations. It remains to be seen whether our findings would be consistent in different populations. Last but not least, the number of samples is not enough and not comprehensive. Due to the exclusion criteria and the limited number of inflammatory cytokines included in the previous GWAS, not all inflammatory cytokines were analyzed in our MR analysis.

In conclusion, a life-long increase in circulating levels of IL-13, as well as lower levels of bFGF and SCF, was associated with a higher risk of ACLF. However, our MR study did not find evidence that genetically predicted ACLF is causally associated with systemic inflammation. As a result of our findings, we demonstrate that cytokines play a crucial role in ACLF pathogenesis. The possibility of using these biomarkers for ACLF prevention and treatment warrants further investigation.

## Data Availability

The original contributions presented in the study are included in the article/Supplementary Material, further inquiries can be directed to the corresponding authors.

## Abbreviations

ACLF, Acute-on-chronic liver failure; MR, Mendelian randomization; GWAS, A genome-wide association study; IVW, Inverse variance weighted; SNP, Single nucleotide polymorphism; SCF, Stem cell factor; bFGF, Basic fibroblast growth factor; IL-13, Interleukin-13; WBC, White blood cell; CRP, C-reactive protein; IVs, Instrument variables.
