# Supplementary material for: Systemic inflammatory regulators and risk of acute-on-chronic liver failure: A bidirectional mendelian-randomization study
Source: Front Cell Dev Biol. 2023 Jan 19;11:1125233. doi: 10.3389/fcell.2023.1125233 (PMC9892464; doi:10.3389/fcell.2023.1125233)
Supplement: Supplementary file 4 [file DataSheet1.docx]

Supplementary Material

Systemic inflammatory regulators and risk of Acute‑on‑chronic Liver failure: a bidirectional Mendelian-randomization study

Shengnan Wang^1†^, Hao Zhu^2†^, Lin Pan^3^, Rui Hua^2#, *^, Pujun Gao^2#, *^

**†These authors have contributed equally to this work and share first authorship**

**^#^ These authors contributed equally to this work and share last authorship**

*** Correspondence:** Corresponding Author: gpj@jlu.edu.cn

# Supplementary Tables

**Supplementary Table S1.** Characteristics of the genetic instrument variables for the cytokines in the Mendelian randomization study at the genome-wide significance level (P < 5 × 10^–8^).

**Supplementary Table S2.** **The Association Between Genetically Predicted Systemic Inflammatory Regulators and ACLF. Sheet1:** Characteristics of the genetic instrument variables for the cytokines in the Mendelian randomization study at level P < 5 × 10^–6^. **Sheet 2:** MR analysis of 41 cytokines and ACLF risk. **Sheet 3:** Heterogeneity analysis of 41 cytokines and ACLF risk. **Sheet 4:** MR Egger intercept analysis of the association between 41 cytokines and ACLF risk. **Sheet 5:** MR PRESSO analysis of the association between 41 cytokines and ACLF risk.

**Supplementary Table S3.** **The Association Between Genetically Predicted ACLF and Systemic Inflammatory Regulator Levels. Sheet1:** Characteristics of the genetic instrument variables for ACLF in the Mendelian randomization study at level P < 5 × 10^–6^. **Sheet 2:** MR analysis of genetically predicted ACLF and 41 cytokine levels. **Sheet 3:** Heterogeneity analysis of genetically predicted ACLF and 41 cytokine levels. **Sheet 4:** MR Egger intercept analysis of the genetically predicted ACLF and 41 cytokine levels. **Sheet 5:** MR PRESSO analysis of the association between genetically predicted ACLF and 41 cytokine levels.

## Supplementary Figures


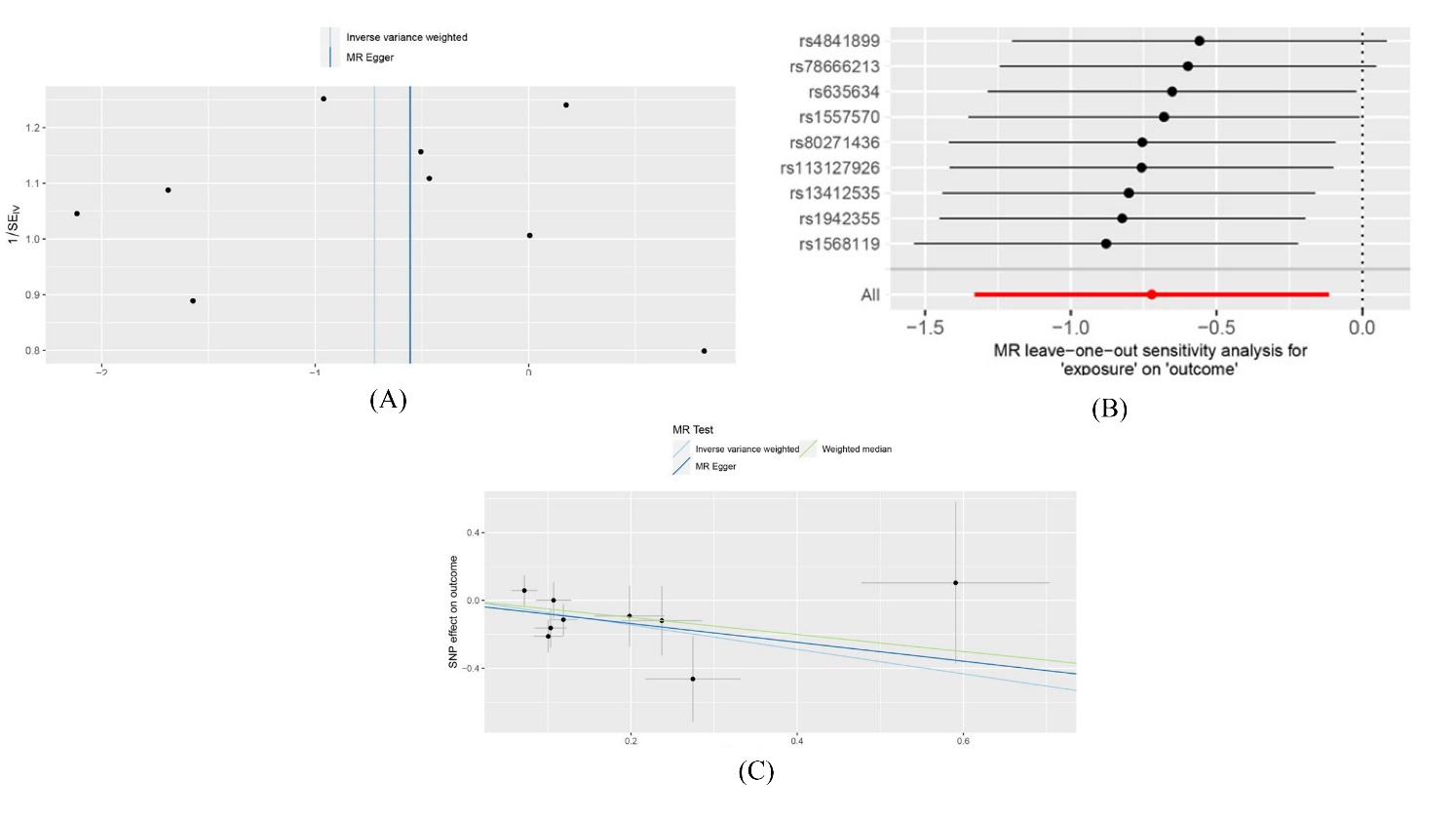


**Supplementary Figure 1.** (A) funnel plots (B) leave-one-out plots (C) scatter plots for the exposure of SCF.


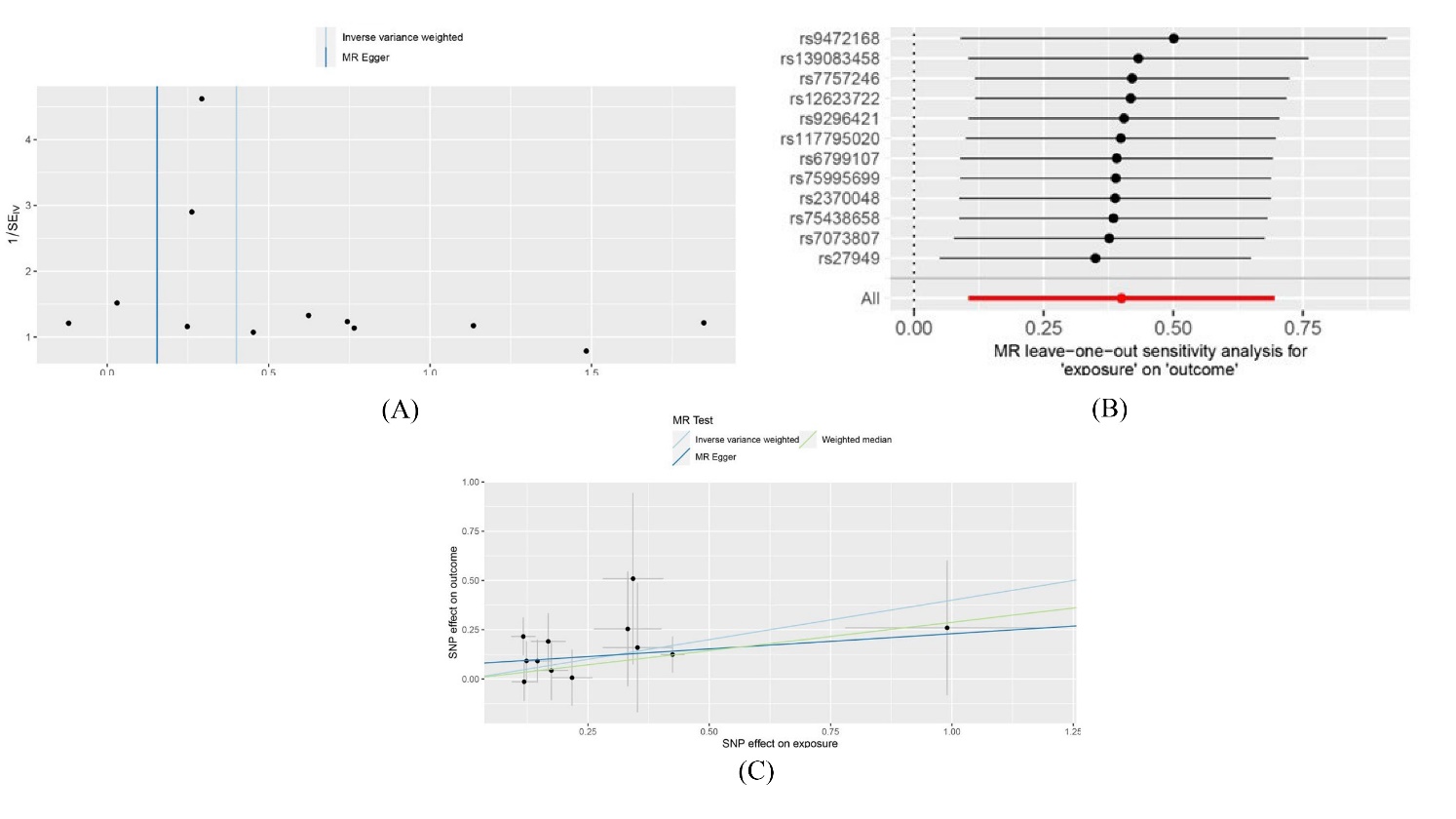


**Supplementary Figure 2.** (A) funnel plots (B) leave-one-out plots (C) scatter plots for the exposure of IL-13.


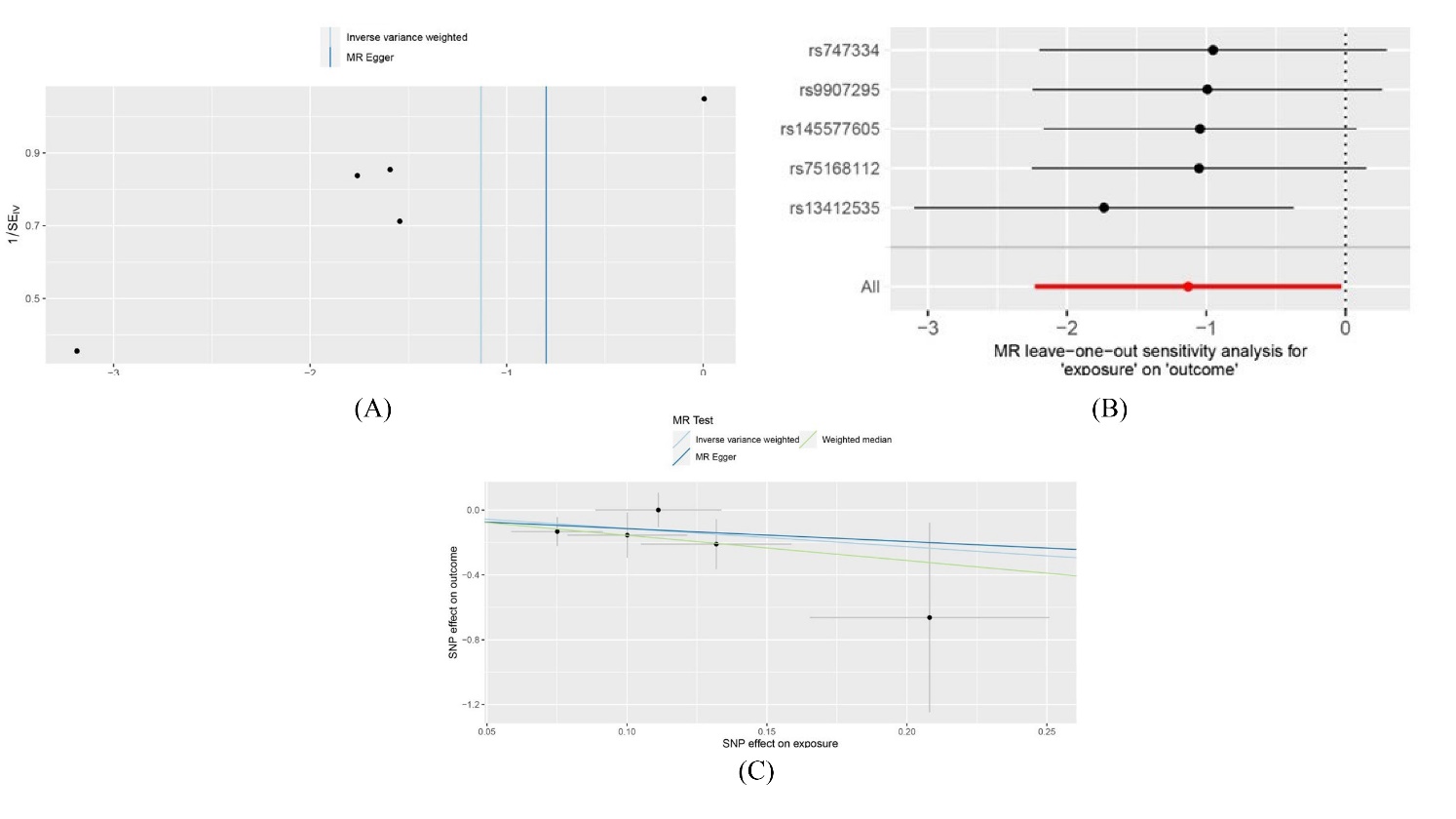


**Supplementary Figure 3.** (A) funnel plots (B) leave-one-out plots (C) scatter plots for the exposure of bFGF.
